# Blood Interferon Signatures Putatively Link Lack of Protection Conferred by the RTS,S Recombinant Malaria Vaccine to an Antigen-specific IgE Response

**DOI:** 10.12688/f1000research.7093.2

**Published:** 2017-07-18

**Authors:** Darawan Rinchai, Scott Presnell, Marta Vidal, Sheetij Dutta, Virander Chauhan, David Cavanagh, Gemma Moncunill, Carlota Dobaño, Damien Chaussabel

**Affiliations:** 1 Sidra Medical and Research Center, Doha, Qatar; 2 Benaroya Research Institute, Seattle, WA, USA; 3 ISGlobal, Barcelona Ctr. Int. Health Res. (CRESIB), Hospital Clínic-Universitat de Barcelona, Barcelona, Catalonia, Spain; 4 Structural Vaccinology Laboratory, Malaria Vaccine Branch, Walter Reed Army Institute of Research, 503 Robert Grant Avenue, Silver Spring, Maryland, 20910, USA; 5 Malaria Research Group, , International Centre for Genetic Engineering and Biotechnology, Aruna Asaf Ali Marg, New Delhi, India; 6 Institute of Immunology & Infection Research and Centre for Immunity, Infection & Evolution, Ashworth Laboratories, School of Biological Sciences, University of Edinburgh, Edinburgh, EH9 3FL, UK

**Keywords:** Transcriptome, Blood, Malaria, Vaccination, IgE, Humoral, Bioinformatics, Modular repertoires

## Abstract

Malaria remains a major cause of mortality and morbidity worldwide. Progress has been made in recent years with the development of vaccines that could pave the way towards protection of hundreds of millions of exposed individuals. Here we used a modular repertoire approach to re-analyze a publically available microarray blood transcriptome dataset monitoring the response to malaria vaccination. We report the seminal identification of interferon signatures in the blood of subjects on days 1, 3 and 14 following administration of the third dose of the RTS,S recombinant malaria vaccine. These signatures at day 1 correlate with protection, and at days 3 and 14 to susceptibility to subsequent challenge of study subjects with live parasites. In addition we putatively link the decreased abundance of interferon-inducible transcripts observed at days 3 and 14 post-vaccination with the elicitation of an antigen-specific IgE response in a subset of vaccine recipients that failed to be protected by the RTS,S vaccine. Furthermore, profiling of antigen-specific levels of IgE in a Mozambican cohort of malaria-exposed children vaccinated with RTS,S identified an association between elevated baseline IgE levels and subsequent development of naturally acquired malaria infection during follow up. Taken together these findings warrant further investigation of the role of antigen-specific IgE in conferring susceptibility to malaria infection.

## Introduction

About 3.4 billion people, nearly half of the world’s population, live in areas at risk of *Plasmodium* transmission ^1^. Malaria infection resulted in an estimated range 149–303 million cases in 2015 that may have caused between 236,000 and 635,000 deaths, according to the World Health Organization ^2^. Recent concerns have been caused by the rise in parasite resistance to artemisinin, which is the last effective monotherapy available for the treatment of malaria ^3^. Cases of artemisinin resistance have been reported from much of Southeast Asia and now appear likely to reach the Indian subcontinent, with potentially dire consequences ^4^. However, significant advances over the past years have been made towards the development of an effective malaria vaccine ^5^. Most notably this includes successful testing of a live vaccine consisting of radiation-attenuated sporozoites ^6^, and this year licensure by regulatory authorities of the first malaria vaccine, the recombinant adjuvanted vaccine developed by global pharmaceutical GSK, called RTS,S also known by its commercial name, Mosquirix ^®^. This is a highly significant landmark but unfortunately the efficacy of the vaccine for unknown reasons, and despite optimization attempts, remains suboptimal ^7, 8^. Thus identification of mechanisms underlying protection conferred by this vaccine, or lack thereof, may be key to the development of a broadly effective prophylactic vaccine against malaria. Unbiased “systems approaches”, consisting in profiling all the elements constitutive of a given biological system, have recently been implemented to investigate responses to vaccines ^9^. Such an approach consisting in measuring blood transcript abundance on a genome-wide scale has been adopted for the serial profiling of responses to the influenza, pneumococcal, yellow fever or malaria vaccines ^10– 15^. In 2010, Vahey *et al.* reported results from a study investigating changes in transcript abundance in blood following administration of the malaria RTS,S vaccine ^15^. In this report we share the results of a re-analysis of the data made available by Vahey *et al.* upon publication of their findings. We employed an approach which consists in identification of modular transcriptional repertoires – collections of co-clustered gene sets – and subsequent modular-level “fingerprinting analyses” ^16^. This re-analysis led to original findings, with the identification of an interferon transcriptional signature at day 1 post-vaccination, correlating with protection as well as a second interferon signature at days 3 and 14 post-vaccination correlating this time with lack of protection of study subjects from subsequent challenges with the malaria parasite. Downstream investigations identified a potential role for IgE responses of restricted specificity in mediating susceptibility to malaria infection.

## Methods

### Construction of the modular repertoire framework

The methodology for constructing modular transcriptional repertoires has been described earlier ^16, 17^. The particular framework employed in this re-analysis has been described in an earlier study investigating responses to influenza and pneumococcal vaccines ^12^. Briefly, nine datasets were used as input, including blood transcriptome profiles generated from patients with HIV, tuberculosis, sepsis, systemic lupus erythematosus, systemic arthritis, and liver transplant. Each dataset was clustered independently using Hartigan’s k-means clustering, using the elbow criterion to determine the optimal number of clusters for each dataset. Cluster membership information for each gene across the nine datasets was used to build a table recording the number of co-clustering events for each possible gene pair. This table was used in turn to build a weighted co-clustering network where each node is a gene and edges indicate co-clustering events with weight ranging from 1 (pair of genes belonging to the same cluster in 1 out of 9 datasets) to 9 (pair of genes belonging to the same cluster in 9 out of 9 datasets). The module selection process consisted in the identification within this large network of cliques, which are densely connected subnetworks. A principled approach was used starting in the first round with the selection of the largest subnetworks carrying the highest weight (co-clustering in 9 out of 9 datasets; corresponding to the M1 modules), followed by identification and removal from the selection pool of the next largest subnetwork and so on (with minimum clique size set at 10). When no additional modules could be identified for a given round of selection the stringency of the selection criteria was progressively relaxed (e.g. co-clustering occurring for 8 out of 9 datasets in the second round of selection, corresponding to the M2 modules; in 7 out of 9 datasets in the third round of selection, corresponding to the M3 modules, etc…). The datasets used for module construction have been deposited in NCBI’s Gene Expression Omnibus: GSE30101. These datasets span a broad range of pathologies, which are associated with inflammation, autoimmunity, infection, cancer, genetic defects of immunity or transplantation, thus allowing the capture of a broad repertoire of immune processes occurring *in vivo*.

### Functional characterization of the blood modular repertoire

Functional analyses were carried out systematically for each module using commercial as well as publically available tools (primarily MetaCore™ version 5.0 and DAVID version 6.7 ^18^) and results are reported on a wiki page: http://www.biir.net/public_wikis/module_annotation/V2_Trial_8_Modules. A complete list of the genes forming the modules is also available from the wiki.

### Module-level analyses

The top six rounds of modules defined by this approach (M1–M6, a total of 62 modules) were used as a framework to analyze and interpret the datasets generated in the context of the Vahey *et al.* study: i.e. rather than carrying out analyses at the individual gene level, which assume that changes in transcript abundance for each gene occur independently from that of other genes, we performed analyses at the modular level, where changes are assessed for sets of co-clustered genes. Thus we summarize “modular response” as a single value, the percent of responsive genes for a given module. In earlier analyses the average fold change per module was also used to demonstrate that high level of concordance could be observed across microarray platforms at the modular level but not at the gene level ^17^. For determining changes for individual subjects post-vaccination a cutoff is set against which individual genes constitutive of a module are tested. If the gene meets the set criteria it is considered “responsive”. “Module-level” data is subsequently expressed as a % value representing the proportion of responsive transcripts for a given module.

***Serum sample for IgE detection.*** IgE levels were measured in serum samples obtained from subjects enrolled in a Phase IIb randomized controlled trial of the RTS,S/AS02 vaccine (ClinicalTrials.gov registry number NCT00197041) previously described by Alonso PL., *et al* ^19– 21^. Mozambican children between 1 and 4 years of age received either the adjuvanted RTS,S vaccine or a comparator vaccine (Hepatitis B vaccines in children older than 24 months; Pneumoccocal and Haemophilus vaccines in younger children). Briefly, blood samples were collected at baseline from children before receiving the vaccine (pre-vaccination) and 1 month after 3 ^rd^ dose of vaccination (post-vaccination). The children who developed malaria infection during follow up were designated as “Non-protected” and those who did not develop infection were designated as “Protected/ Non exposed”. **Cohort 1**: “Non-protected” were defined as children who had an episode of clinical malaria determined by passive case detection. That means that only sick children going to a health center with malaria diagnosed by blood smear were considered “Non-protected”. “Protected/ Non exposed” were children who did not have any malaria episode during follow up, but they could have had an infection but did not go to a health post. **Cohort 2:** “Non-protected” were defined as children who had a *P. falciparum* infection determined by passive and active case detection. The difference with the cohort 1 was that every 15 days for the first 2.5 months and monthly after that, subjects had visits at home to check temperature and if they had any fever then a slide and filter paper for PCR to detect parasitemia were done. Therefore, in this cohort it was more unlikely if a child had an infection that it would go unnoticed. “Protected/ Non exposed” were children who did not have any *P. falciparum* infection during follow up. Cohort 1 was from a medium transmission intensity area whereas Cohort 2 was from a high transmission intensity area. The study was approved by the Mozambican National Health and Bioethics Committee (Ref. 237/CNBS), the Hospital Clínic of Barcelona Ethics Committee (Registro 2008/4444), and the PATH Research Ethics Committee (Study file number HS 482) and written informed consent was gathered from parents/guardians ^19, 20^.

***IgE multiplex assay.*** Total and antigen-specific IgE levels were measured using a suspension array technology. Serum samples of 23 children who received comparator vaccines (11 Non-protected and 12 Protected/Non-exposed subjects) and 44 children who received the RTS, S vaccine (22 Non-protected and 22 Protected/Non-exposed subjects) were assessed in this assay, together with positive and negative controls. Each plate was designed balancing samples from both groups of vaccination, protection status, both cohorts, age and sex.

To measure total IgE levels, anti-human IgE-coupled beads (MAGPlex Beads, Luminex Corp.) were mixed with serum samples at 1/50 dilution to capture total IgE. Biotinilated anti-human IgE followed by streptavidin-phycoerythrin were added to detect IgE. An 18 points-Standard curve starting at 2000 ng/ml of human IgE and finishing at 0.015 ng/ml was included as part of quality assurance/quality control (QA/QC).

To measure RTS,S antigen-specific IgE levels, six *Plasmodium falciparum* antigens and one hepatitis B virus antigen were coupled to different bead populations to create a multiplex panel: circumsporozoite surface protein (full length CSP, purchased from Sanaria), NANP repeat region of CSP and C-term part of CSP (obtained from Walter Reed Army Institute of Research - WRAIR, USA), MSP2 (from 3D7 strain, obtained from the Institute of Immunology & Infection Research and Centre for Immunity, Infection & Evolution, University of Edinburgh, UK), MSP3 (from 3D7 strain, obtained from the International Centre for Genetic Engineering and Biotechnology, India), EXP1 (purchased from Sanaria) and hepatitis B surface antigen (HBsAg, purchased from Abcam). Because CSP NANP repeat, C-term fragments and MSP2 were expressed with a GST-Tag, a GST-coupled bead was also included. Briefly, antigen-coupled beads were mixed with serum samples at dilutions 1/30, 1/270, 1/2,430 and 1/21,870 to capture antigen-specific IgE. Subsequently, mouse anti-human IgE and biotinilated goat anti-mouse IgG were added as secondary and tertiary antibodies and finally streptavidin-phycoerythrin was added. Results are expressed in Median Fluorescence Intensity (MFI) and at least 50 beads per analyte/well were acquired in Luminex xMAP® 100/200 analyzer.

### Statistical analyses

Mann Whitney tests were performed on individual module response values expressed as percentages comparing protected and non-protected groups. Wilcoxon signed-rank test were performed to compare response between pre-vaccination and post-vaccination using GraphPad Prism software version 6 (GraphPad Software, San Diego, CA).

## Results

### Study design and reanalysis using modular repertoires

The design of the vaccine trial is described in detail by Vahey *et al.* and in an earlier publication ^15, 22^. Briefly, study subjects received the RTS,S vaccine, which consists of sequences of the *P. falciparum* CSP expressed in hepatitis B surface antigen and formulated with the proprietary adjuvant systems AS01/AS02 ^23^. Challenge was performed with a homologous 3D7 strain of *P. falciparum* delivered by 5 bites from infected mosquitoes. Samples were obtained from study participants at study entry (36 samples); on the day of the third vaccination (44 samples); at day 1 (43 samples), day 3 (43 samples), and day 14 (37 samples) thereafter; and at day 5 post-challenge (39 samples). Peripheral blood mononuclear cells (PBMC) transcriptome profiles were generated using commercial Affymetrix HG-U133 chips. Data processing and normalization methodologies are described in the original publication. Data are available publically from the NCBI Gene Expression Omnibus ( GSE18323). Only the blood transcriptional profiles generated on the day of the third vaccination and at day 1, day 3 and day 14 post-third vaccination were used in our re-analysis.

We employed a “modular repertoire approach” first described in 2008 in a research paper ^17^, and more recently in a review ^16^. Briefly, this approach consists in a priori identifying relationships among constituents of a given biological system, which in our case is the blood transcriptome. This makes it in turn possible to analyze transcriptional profiles as functionally interpretable gene sets rather than independent genes. Modular repertoires are established in an entirely data-driven process through the recording of co-clustering patterns of transcripts across a wide range of immune-related diseases. A collection of datasets encompassing infectious as well as autoimmune disorders and primary immune deficiency was used as input in order to capture a wide variety of immune signatures. The module construction process and modular analyses are described in detail in the Methods section.

In the original analysis of this dataset Vahey *et al.* report the identification: 1) of a transient signature at 24 hours post-vaccination that was not observed at subsequent time points. This signature is described as being associated with inflammatory processes elicited by the vaccine and was not associated with outcome of the infectious challenge; 2) of a signature at 5 days post-challenge that distinguish vaccinated from non-vaccinated individuals, thus directly reflecting and demonstrating the effect of vaccination; 3) of a signature at 14 days post-vaccination correlating with protection conferred by the vaccine. This 393-gene signature was identified using high resolution Gene Set Enrichment Analysis (GSEA) and consisted in transcripts belonging to the immunoproteasome pathway associated with the processing of major histocompatibility complex class I peptides.

### Increased interferon module response at day 1 correlates with protection

**Figure 1.**
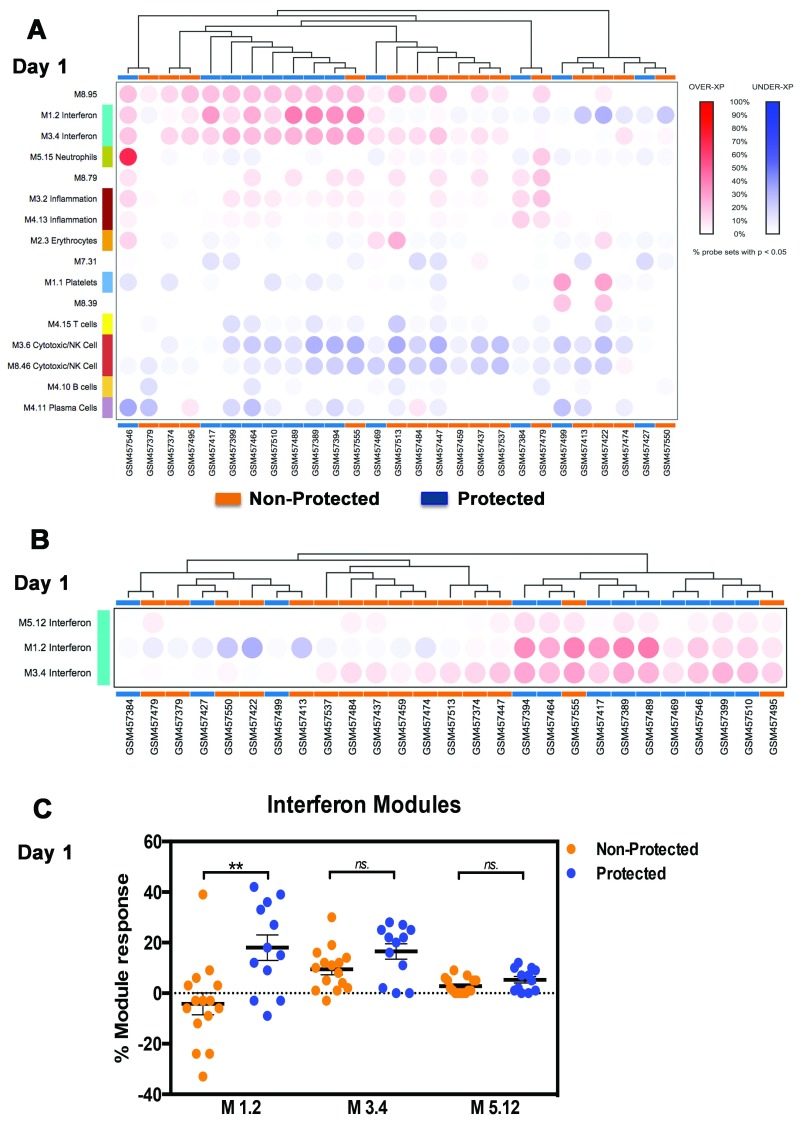
Increased modular interferon response at day 1. Blood transcriptional responsiveness to malaria vaccination was determined at the modular level in subjects one day following administration of the third dose of RTS,S. **A.** The percentage of responsive transcripts was determined for each module (co-clustered gene set) and represented by a colored spot on a heatmap where modules are arranged in rows and samples in columns (using a custom web; manuscript describing this resource is in preparation). Increases in transcript abundance compared to baseline pre-third vaccine sample are shown in red and decreases in transcript abundance in blue. Modules and samples are arranged by hierarchical clustering based on patterns of module responsiveness. **B.** Grouping of samples based on patterns of responsiveness of interferon modules is shown here. **C.** Responsiveness of the three interferon modules on day 1 is shown on a plot.

In our re-analysis we first assessed changes in transcript abundance at the modular level. The percentage of responsive transcripts constitutive of a given module was determined for each individual at days 1, 3 and 14 following administration of the third vaccine dose in comparison to the levels obtained in samples collected just prior to that injection (see Methods for details). Hierarchical clustering was then performed at each time point to group modules (rows) and subjects (columns) based on patterns of changes in blood transcript abundance represented by the percent module response values (day 1, Figure 1A). Modules were filtered to only retain those with changes >15% in at least one subject. This arbitrary cutoff is set at three times the false discovery rate used for multiple testing correction (5%). This analysis is unsupervised since it does not take knowledge of outcome of the infectious challenge into account. We observed nonetheless that samples tended to segregate based on whether or not the vaccine conferred protection ( Figure 1A). Three modules associated with induction by interferon appeared to be the main elements driving the clustering of study subjects, with higher abundance levels being observed in subjects protected from subsequent infectious challenge. We demonstrated in our previous work that those three interferon modules represent distinct signatures that can be used for stratification of subjects with systemic lupus erythematosus ^24^. Thus, we used in turn the same M1.2, M3.4 and M5.12 modules to stratify malaria vaccine recipients. Hierarchical clustering using only this subset of modules contributed to further separation of subjects based on the outcome of the infectious challenge ( Figure 1B). The difference in % module responsiveness between protected and non-protected subjects was also statistically significant for M1.2 (p=0.0094, Mann Whitney test) ( Figure 1C). M3.4 tended to be elevated compared to pre-vaccination baseline in both protected and non-protected individuals but was not different between those two groups. Abundance of M5.12 transcripts did not change following vaccination.

### Decreased interferon module response at days 3 and 14 correlate with lack of protection

**Figure 2.**
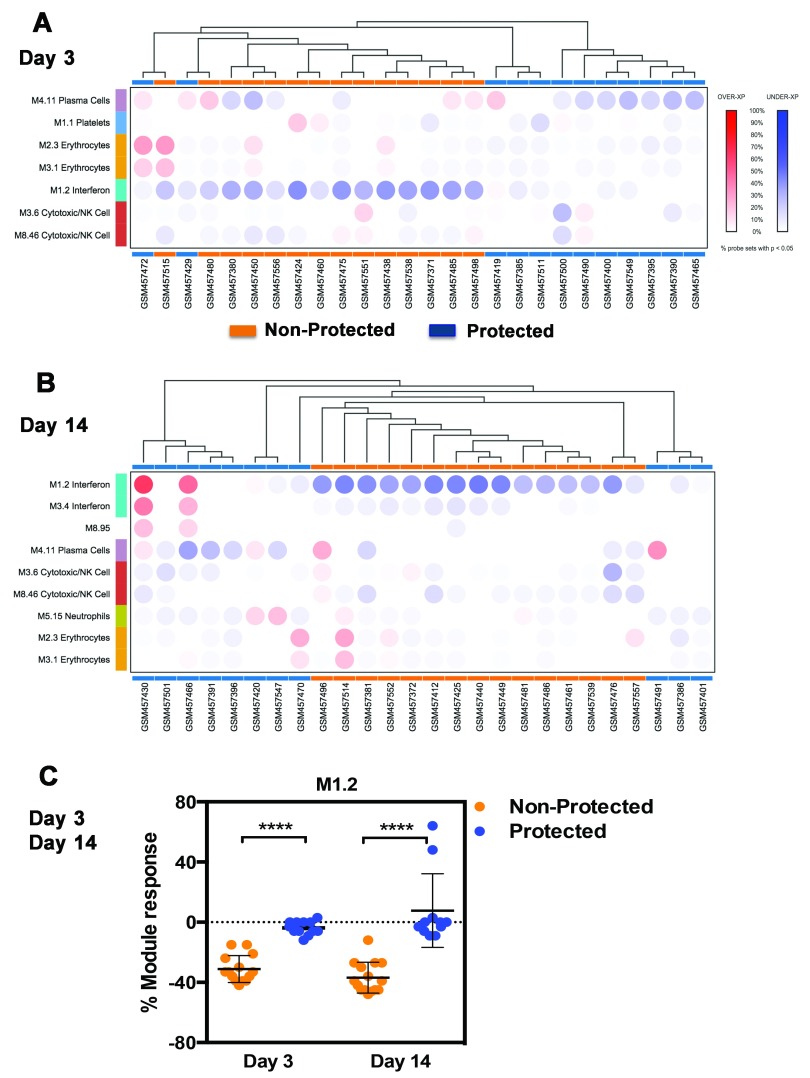
Decreased modular interferon response at days 3 and 14. Blood transcriptional responsiveness to malaria vaccination was determined at the modular level in subjects 3 and 14 days following administration of the third dose of RTS,S. **A.** As is the case of Figure 1 the percentage of responsive transcripts was determined for each module (co-clustered gene set) and represented by a colored spot on a heatmap where modules are arranged in rows and samples in columns. Increases in transcript abundance compared to baseline pre-third vaccine sample are shown in red and decreases in transcript abundance in blue. Modules and samples are arranged by hierarchical clustering based on patterns of module responsiveness. **B.** Similar description as in **A**, this time applied to day 14 data. **C.** Responsiveness of module M1.2 at days 3 and 14 post vaccination are represented on a plot showing also differences between the protected and non-protected groups.

We next used a similar approach to classify subjects at days 3 and 14 post-third vaccination. Subjects once again segregated based on whether or not protection is conferred by the vaccine ( Figures 2A & 2B). Notably, however, at these time points the signature showed a decrease in levels of transcript abundance in comparison to baseline pre-vaccine samples in subjects that were not protected. Thus conversely with the signature described at day 1, signatures at days 3 and 14 correlated with lack of protection by the vaccine. Differences between protected and non-protected groups where highly significant for M1.2 ( Figure 2C, day 3 p<0.0001, day 14 p<0.0001, Mann Whitney test). M3.4 and M5.12 did not show significant differences between those groups. Notably, we found that the genes constitutive of M1.2, M3.4 and M5.12 do not overlap with the day 14 immunoproteasome signature (32 genes) described by Vahey *et al* ( Supplementary figure 1). All but three of the 32 genes mapped to the modules constituting our repertoire. Six genes mapped to module M5.13 and three to module M5.4, the remaining 23 genes mapped to 18 other modules. However, none of the genes mapped to either of the three interferon modules identified as associated with protection by RTS,S vaccine at Day 1, 3 or 14 after the third vaccination. Notably, IFNG, which was part of the 32 gene signature is among the genes constituting M3.6, which is associated with NK cells/Cytotoxic activity. Taken together results of our reanalysis of the Vahey dataset using a modular repertoire framework led to an original finding, by demonstrating the association between diverging day 1 and days 3 and 14 interferon signatures and protection conferred by the RTS,S vaccine.

### Putative model accounting for the contrasted interferon signatures observed in response to RTS,S vaccination

We have shown in an earlier work that the three interferon modules that were described above tend to become elevated sequentially in patients with systemic lupus and may be associated with differential induction of type I and type II interferon in this disease ^24^. Furthermore, lupus disease severity was found to correlate significantly with M5.12 levels. We have also shown that an interferon response dominated by M1.2 and M3.4 was transiently increased 1 day following vaccination with the trivalent influenza virus ^12^. O’Gorman *et al.* recently demonstrated that this transient interferon response is mediated by flu antigen-specific IgG immune complexes rather than engagement of pathogen-associated molecular pattern receptors ^25^. The day 1 interferon response observed in the context of malaria vaccination could similarly be the result of engagement of CSP-specific IgG immune complexes since it occurs following administration of the third dose of RTS,S, at a time when a pre-existing humoral response would have been elicited by the first two doses.

But most peculiar is the fact that this increased modular interferon response in protected individuals at day 1 is followed by a persistent decrease in abundance of M1.2 transcripts below the pre-vaccination baseline in individuals who were not protected by the RTS,S vaccine. Indeed, in over 10 years of investigating blood transcriptome responses in a wide range of clinical and experimental settings the authors have not encountered a single instance of such a sustained and uniform decrease in abundance of interferon-inducible transcript. What is especially striking is the clear cut association between lack of protection conferred by RTS,S with the decrease in abundance of M1.2 transcripts seen in Figure 2C. This implies that the immunological mechanism underlying this suppressed interferon signature may be key to overcoming current limitations of sub-unit malaria vaccination.

**Figure 3.**
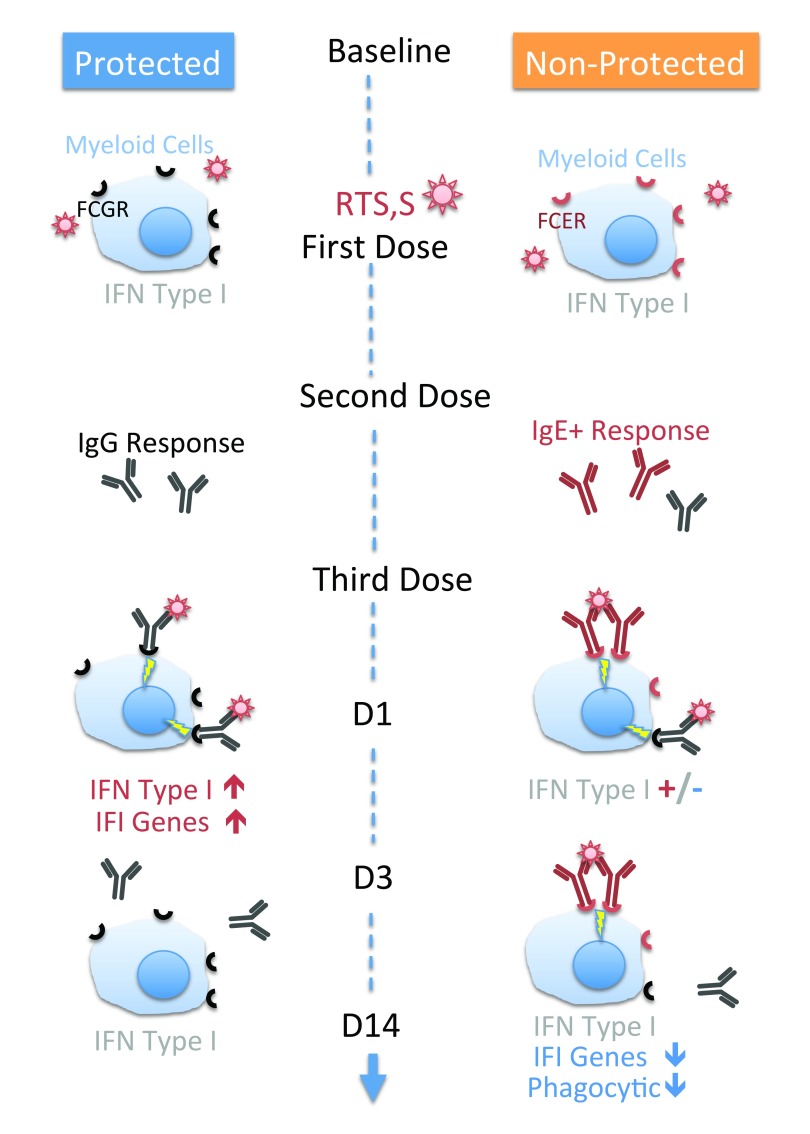
Proposed immunological mechanism determining protection – or lack thereof – in adult human individuals to whom was inoculated the RTS,S vaccine. Our model infers that the interferon signatures observed on days 1, 3 and 14 post-vaccination correlating with outcome of the processes that deploy post the co-delivery of Anopheles saliva and *P. falciparum* sporozoites, are the result of engagement of Fc receptors by immune complexes. According to our model no interferon signatures should be observed following administration of the first vaccine dose of the CSP- containing RTS,S vaccine, in absence of pre-existing immune effectors/regulators reactive to *P. falciparum* Circum Sporozoite Protein/CSP. The injection of the first two doses of vaccines should elicit a humoral response, which in non-protected individuals is dominated by CSP- binding IgE rather than IgG. CSP- Ig- immune complexes should form when the third vaccine dose is administered. The transient interferon response elicited in individuals who develop a protective response and that we observed at Day 1 could be mediated by engagement of the FcγR by IgG-CSP immune complexes as has been described earlier in the context of influenza vaccination) ^25^. Our model predicts that IgE-CSP complexes form in non-protected individuals and cross-link the high affinity IgE receptor at the surface of cells of the myeloid lineage. FCER1 engagement would in turn mediate reduction in levels of IFN-inducible transcripts that is observed on days 3 and 14. This reduction of constitutive levels of IFN-inducible transcripts in the non-protected group would be countered at least partially on Day 1 by residual IgG-IC response in subjects displaying mixed IgE/IgG humoral responses. If this is indeed the case the levels of M1.2 reduction on Day 1 in individuals displaying CSP-binding IgE should be inversely correlated with levels of CSP-specific IgG. Given the transient nature of the interferon signature that was observed and that we tentatively attribute to IgG ICs, this partial reversal of IgE mediated suppression would not be observed at later time point, which would account for the sustained decrease in abundance of interferon-inducible transcripts that was observed at days 3 and 14. Engagement and crosslinking of FCER1 may also result in altered phagocytic and parasite killing abilities ^33^ thus potentially contributing to diminished resistance to infection in cases where the humoral response to RTS,S vaccination is dominated by IgE.

**Figure 4.**
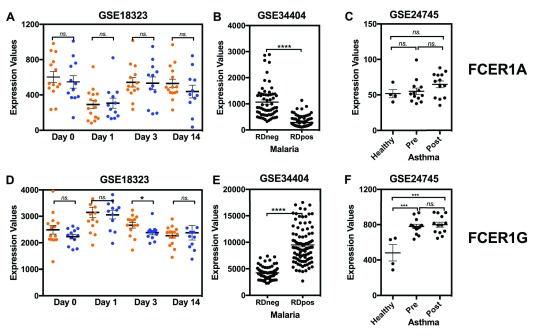
Abundance of FCER1A and FCER1G transcripts in whole blood samples of RTS,S vaccinated subjects, acute malaria infection and asthma patients. mRNA expression levels for FCER1A and FCER1G were measured by microarrays in whole blood obtained following RTS,S vaccination (GSE18323), or in patients with acute malaria infection (GSE34404) or asthma (GSE2475). **GSE18323:** Samples were collected at study entry, the day of third vaccination (Day 0), 24 (Day 1) and 72 (Day 3) hours post the third vaccination and two weeks (Day14) post the third vaccination. Of 39 vaccine recipients, 13 were protected (blue) and 26 were not protected (orange) ^15^. https://www.ncbi.nlm.nih.gov/geo/query/acc.cgi?acc=GSE18323. **GSE34404:** Whole blood gene expression profiles and genotypes from 155 West-African children including 94 cases (Rapid Diagnostic (RD) Positive) undergoing the symptomatic phase of blood-stage *P. falciparum* infection and 61 age-matched controls (RD negative) ^34^. https://www.ncbi.nlm.nih.gov/geo/query/acc.cgi?acc=GSE34404. **GSE24745:** Asthmatic subjects (20–60 years of age, with stable, mild allergic asthma, n=17) underwent allergen inhalation challenges. All subjects had an early asthmatic response of ≥ 20% fall in FEV1; most had a late phase response of ≥ 15% fall in FEV1. Blood was collected immediately prior to, and two hours after allergen challenge ^35^. https://www.ncbi.nlm.nih.gov/geo/query/acc.cgi?acc=GSE24745.

Here we putatively attribute this decrease in abundance of interferon-inducible transcripts and subsequent lack of protection to the elicitation by the vaccine of an antigen-specific IgE response. This hypothesis is based on an array of converging evidence:

1) Engagement of the high affinity IgE receptor, FCER1, mediates decreased responsiveness to interferon-inducing stimuli. Gill *et al.* have shown that plasmacytoid dendritic cells (pDCs) isolated from patients with allergic asthma produce reduced levels of interferon alpha in response to the influenza virus *in vitro* when compared with pDCs isolated from non-asthmatic controls ^26^. They also demonstrated that production of interferon alpha by pDC stimulated *in vitro* with the virus is significantly decreased upon cross-linking of the FCER1 receptor ^26^. Similar findings have been reported more recently in PBMCs exposed to Human Rhinovirus (HRV) ^27^. This is to our knowledge the only immune-mediated mechanisms of suppression of interferon responses that may explain the decrease in M1.2 observed following RTS,S vaccination. Thus we hypothesize that the suppression by RTS,S of levels of interferon inducible transcripts results from formation of IgE-CSP immune complexes, with anti-CSP IgE being elicited in earlier rounds of vaccination ( Figure 3). An alternative explanation is advanced by one of the reviewers, Dr Luty, in his comments, who mentions the potential relevance of the use of a 226 amino acid stretch of HBsAg as a conjugate for the CSP antigen in the RTS,S vaccine. Indeed this molecule has also been shown to inhibit Toll-like Receptor (TLR)9-mediated IFN-α production by pDC ^28^. and “could thus potentially be exerting some influence on the outcome of immunization in term of the anti-malarial protection generated” ^29^. This is indeed another possibility, which warrants testing of the inhibitory capacity of the protein fragment used in RTS,S vaccine formulation. Furthermore the cell populations and signaling pathways involved in elicitation and modulation of interferon responses by RTS,S vaccine would also need to be investigated. Indeed, as mentioned earlier it was found for instance that, rather unexpectedly, the induction of interferon response by the trivalent influenza vaccine is mediated by IgG immune-complexes rather than TLR engagement ^25^. We hypothesized that interferon signatures observed in response to RTS,S vaccination arise through a similar mechanism, which may be antagonized by IgE immune complexes.
2) Further evidence pointing to the influence of IgE responses comes from the literature and from results of our preliminary investigation reported below showing a dependence on both pre-erythrocytic and blood stages and antigen specificity of IgE on outcomes of malaria infection in children from endemic areas. Perlmann *et al.* identified IgE as a pathogenic factor in malaria, with immune complexes contributing to excess TNF induction in PBMC *in vitro* ^30^. Mice deficient for the high affinity IgE receptor have shown increased resistance to blood-stage of parasite infection, specifically implicating FCER1 expressing neutrophils as pathogenic mediators ^31^. A more recent study has established a link between asthma and atopic dermatitis and delayed development of clinical immunity to *P. falciparum* ^32^. Notably, in addition to shifting cytokine balance by promoting IL-10 and TNF production, engagement of high affinity IgE receptors has been reported to critically impair phagocytic function of monocytes, a mechanism that is essential for the control of malaria infection ^33^. The antigen specificity of IgE responses that have been associated with enhanced malaria morbidity remains to be determined. It is of relevance to RTS,S vaccination, at least in naïve subjects, since the vaccine targets the liver stage rather than the blood stage of the parasite.
3) At Dr Luty’s suggestion we also examined levels of abundance of FCER1 subunits transcripts in the RTS,S vaccine dataset used in our analysis ( Figure 4). Consistently with what is observed during acute malaria infection (GSE34404) ^34^, FCER1A and FCER1G levels were respectively increased and decreased one day post-RTS,S vaccination. However, notably, FCER1G levels remained significantly elevated at day 3 post-vaccination in non-protected individuals, while they decreased to baseline levels in protected individuals ( Figure 4D). Abundance of FCER1G transcript was also significantly elevated in a third dataset where changes were measured in patients during episodes of asthma exacerbation (GSE24745) ^35^. Moreover, we also checked expression level of FCGR2A and FCGR2B in the same dataset and found that abundance of FCGR2A and FCGR2B were not different in subjects who were protected and in those who were not protected. Thus, taken together, these additional observations adds to the array of indirect evidence pointing to IgE responses as potential determinants of protection by the RTS,S vaccine.

***Induction of IgE anti-CSP antigens after RTS,S vaccination.*** Based on those findings we contacted Drs. Dobaño and Moncunill from ISGlobal, Barcelona, Spain who previously measured IgG responses to 824 *P. falciparum* antigens by Luminex assay and protein array in Mozambican children 6 months after receiving a full course of RTS,S (n=291) versus comparator vaccine (n=297) in a Phase IIb trial. In order to further investigate the role of IgE responses in the context of RTS,S vaccination a novel Luminex assay was developed for measuring levels of total IgE, as well as IgE specific for CSP antigen (full length CSP, C-term, NANP repeat) and other blood stage vaccine candidate antigens (see methods for details). Pre-vaccinated and post-vaccinated serum samples of 23 children who received comparator vaccines (including 11 subjects who developed clinical malaria during follow up and 12 subjects who did not) and 44 children who received RTS,S vaccine were tested (22 subjects who developed clinical malaria and 22 subjects who did not). In our interpretation of the results of this study we consider that the individuals who developed infection during the follow up period were not protected (noted as “not protected”), while those who did not develop infection were either protected or not exposed (noted as “protected/non-exposed”).

**Figure 5.**
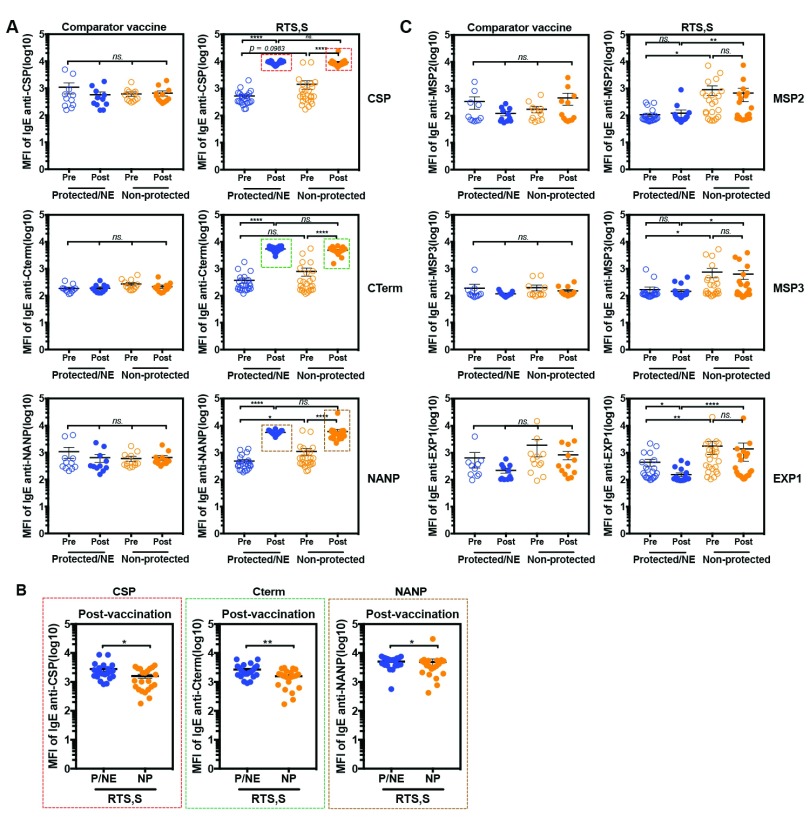
Detection of antigen-specific IgE in serum samples of children who received the RTS,S vaccine or comparator vaccines. IgE levels are shown for 23 children who received the comparator vaccines (11 non-protected (NP) and 12 protected(P)/Non-exposed (NE) subjects) and 44 children who received the RTS,S vaccine (22 non-protected and 22 protected/NT subjects). Blood samples were collected from children at baseline before receiving the vaccine (pre-vaccination) and 1 month after the 3 ^rd^ dose of vaccination (post-vaccination). The children who developed malaria infection during follow up were classified in the “Non-protected” group and those who did not, were classified in the “Protected/NE” group. **A**) comparison of levels of CSP-, CTerm-, NANP-specific IgE (dilution 1:30), and MSP2-, MSP3-, EXP1-specific IgE (dilution 1: 2,430) in serum samples of comparator vaccine control or RTS,S vaccine groups. Red, green and brown boxes identify the samples that were over the higher limit of quantification. **B**) Antigen-specific IgE in serum samples of RTS,S vaccinated group identified by the red, green and brown boxes in panel A which were re-run at a higher dilution (1: 21,870). **C**) Comparison of levels of MSP2-, MSP3- and EXP1-specific IgE in serum samples of comparator vaccine control or RTS,S vaccine; blue represented “Protected/NE” and orange represented “Non-protected”. The results represent means ± standard errors (SE). Statistical significance was determined using Wilcoxon signed-rank test or Mann Whitney test; ns, not significant, * *p < 0.05*, ** *p < 0.01*, *** *p < 0.001* and **** *p* < *0.0001*. The horizontal lines indicate mean ± standard errors (SE).

***Induction of IgE anti-CSP antigens after RTS,S vaccination.*** The results obtained demonstrate, first that RTS,S vaccination elicits a robust IgE response ( Figure 5A). Indeed, levels of CSP-specific IgE were significantly higher in serum samples post-vaccination as compared to pre-vaccination, these results were confirmed when different regions of CSP (C-Term and NANP) and HBsAg were tested.

Second, post-vaccination levels of CSP antigen-specific IgE were not different between the non-protected (NP) and protected (P)/non-exposed (NE) groups at the lower serum dilutions tested ( Figure 5A), and were significantly lower in the non-protected group compared to the protected/NE at higher dilutions ( Figure 5B). This finding does not support our initial hypothesis, which putatively attributed the suppressed interferon signatures observed in non-protected individuals to higher levels of antigen-specific IgE. However, the fact that the subjects in the first study were malaria-naïve adults, and in the second were pre-exposed children does not allow drawing definitive conclusions on this point yet.

**Figure 6.**
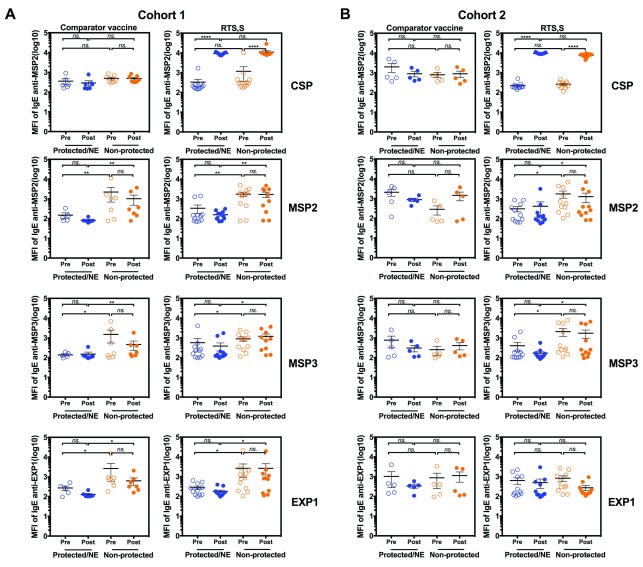
Comparison of specific IgE in serum samples of children from cohort 1 and cohort 2 who received the RTS,S vaccine or comparator vaccines (1:270 dillution). 23 children received comparator vaccines (11 non-protected and 12 protected(P)/Non-exposed (NE) subjects) and 44 children received the RTS,S vaccine (22 non-protected and 22 protected/NE subjects). Blood samples were collected from children at baseline before receiving the vaccine (pre-vaccination) and 1 month after the 3 ^rd^ vaccine dose (post-vaccination). The children who developed malaria infection were classified in the “Non-protected” group and those who did not were in the “Protected/NE” group. The samples were diluted at 1:270 for the IgE assay. **A**) Comparison of CSP-, MSP2-, MSP3- and EXP1-specific IgE levels in serum samples of comparator vaccine control or RTS,S vaccine groups from cohort 1 (low transmission area, passive case detection). **B**) Comparison of levels of CSP-, MSP2-, MSP3- and EXP1-specific IgE levels in serum samples of comparator vaccine control or RTS,S vaccine groups from cohort 2 (high transmission area, active case detection). Blue represents “Protected/NE” and orange represented “Non-protected”. The results represent means ± standard errors (SE). Statistical significance was determined using Wilcoxon signed-rank test or Mann Whitney test; ns, not significant * *p < 0.05*, ** *p < 0.01* and **** *p* < *0.0001*. The horizontal lines indicate mean ± standard errors (SE).

Indeed, thirdly, it was observed that levels of anti-CSP IgE in pre-vaccination samples of the non-protected group tended to be higher than in the protected/ NE group ( Figure 5A). A similar, and more marked trend was observed when measuring total IgE in both comparator and RTS,S vaccines groups ( Supplementary Figure 2). However, notably, levels of IgE specific for MSP2, MSP3 and EXP1 antigens, which are not part of RTS,S vaccine, were significantly elevated in the non-protected group when compared to the protected/NE group in both pre- and post- RTS,S vaccination time points ( Figure 5C). Given that such differences were not observed in the comparator vaccine group we analyzed separately subjects from cohort 1 (low/moderate transmission, passive case detection) and from cohort 2 (high transmission, active case detection). Levels of IgE were significantly higher in non-protected groups of both comparator vaccine control and RTS,S vaccine ( Figure 6 / and Supplementary figure 3.

Altogether these observations suggest that IgE specific antibodies are also produced during natural exposure, and that elevated levels of IgE specific for blood stage antigens may be associated with poor clinical outcomes upon parasite re-exposure, regardless of RTS,S vaccination status.

## Conclusions

Gaining an understanding of immunological mechanisms that confer protection via immunization with the RTS,S malaria vaccine, or conversely that prevent it, can help address decisively the global health challenges caused by malaria infection. In this report we identify an interferon blood transcriptional signature correlating with protection following subsequent infectious challenge. As pointed out in the comments posted by Dr Luty, one of the reviewers, this finding is consistent with immunological profiling results obtained by the team who carried out the trial and who reported higher frequencies of IFN-γ-producing T cells in individuals protected from an experimental challenge ^22, 29^. However, the mechanism of induction of the transcriptional interferon signature that was observed and the nature of the leukocyte population(s) carrying this signature remains to be elucidated in the context of RTS,S vaccination.

Furthermore, we tentatively attributed the peculiar decrease in abundance of interferon-inducible transcripts observed at days 3 and 14 following administration of the third dose of the vaccine to the possible elicitation of an IgE response in a subset of individuals that failed to be protected by vaccination. This hypothesis was corroborated indirectly by the sustained increase in levels of FCER1G transcripts observed in response to vaccination in the non-protected group. As we sought to gather additional evidence we turned to collaborators, Drs Dobaño and Moncunill, in order to test IgE responses in vaccinated pediatric subjects from Mozambique. Notable differences from the original study included age, ethnicity and, importantly, prior exposure to the pathogen since the subjects live in regions where malaria is endemic. Despite the pre-immune status, as was previously reported with IgGs ^36^, we found the RTS,S vaccine to be a potent inducer of IgE responses. We also found that level of CSP-specific IgEs post-vaccination were either not different or lower in non-protected individuals, which goes against our initial hypothesis putatively implicating elevated IgE levels for the suppressed interferon signatures observed on days 3 and 14 post-vaccination. However, given significant differences in the respective settings for the trials in which transcriptional and serological profiles were obtained it is not yet possible to conclude decisively on this point. Indeed, as one of the reviewers, Dr Luty, pointed out in his comments: “the vaccine’s capacity for induction of immunological responses in malaria non-exposed adults in North America may be quite far removed from its capacity to induce responses in sub-Saharan African newborns or infants, many of whom could have had some exposure to the pathogen or its products already either in utero or in early life” ^29^. The potential role played by prior *P. falciparum* exposure is evidenced by the fact that levels of MSP-specific IgE at pre-vaccination baseline was significantly higher in non-protected individuals, compared to protected individuals. A similar trend was observed for CSP-specific IgEs and total IgEs but differences were not significant. Notably, the differences were also more marked in the first cohort of subjects from low to moderate transmission areas for which infections were monitored passively (based on occurrence of clinical symptoms). The observation that IgE levels and specificity resulting from natural exposure to parasite in endemic areas was associated with development of disease may be a finding of potential clinical significance that remains to be replicated in independent studies. Furthermore, as pointed out by Dr Milon in her comments, extending the investigation to include profiling of IgE which are specific for antigen present in the saliva of the vector is also warranted ^37^.

## Supplementary material

Supplementary figure 1

**Mapping of the immunoproteasome geneset to the blood modular repertoire framework.** Thirty two genes constituting the immunoproteasome signature described by Vahey *et al.*, listed on the left panel, were mapped to 260 modules used in our reanalysis. The genes mapped to 20 different modules. Their distribution is shown on the histogram on the right. Three genes which did not map to any of the modules were assigned to the NA group.

Supplementary figure 2

**Detection of total IgE in serum samples of children who received the RTS,S vaccine or comparator vaccines.** 23 children received comparator vaccines (11 non-protected and 12 protected/ Non-exposed (NE) subjects) and 44 children received the RTS,S vaccine (22 non-protected and 22 protected subjects). Blood samples were collected from children at baseline before receiving the vaccine (pre-vaccination) and 1 month after 3 ^rd^ dose of vaccination (post-vaccination). The children who developed clinical malaria are designated as “non-protected” and those who did not develop malaria were designated as protected/NE (protected or non exposed). A) comparison of total IgE in serum samples of comparator vaccine control group pre- and post-vaccination. B) comparison of total IgE in serum samples of RTS,S vaccine group pre- and post-vaccination; blue represented protected/NE subjects and orange represented non-protected subjects. The results represent means ± standard errors (SE) of median fluorescence intensity (MFI). Statistical significance was determined using Wilcoxon signed-rank test or Mann Whitney test ns, not significant and **** *p* < *0.0001*. The horizontal lines indicate mean ± standard errors (SE).

Supplementary figure 3

**Comparison of specific IgE in serum samples of children from cohort 1 and cohort 2 who received the RTS,S vaccine or comparator vaccines (1:2340 dillution).** 23 children received comparator vaccines (11 non-protected and 12 protected(P)/Non-exposed (NE) subjects) and 44 children received the RTS,S vaccine (22 non-protected and 22 protected/NE subjects). Blood was collected from subjects at baseline, before receiving the vaccine (pre-vaccination) and 1 month after 3 ^rd^ dose of vaccination (post-vaccination). The children who developed malaria infection were classified in the “Non-protected” group and those who did not develop infection in the “Protected/NE” group. The samples were diluted at 1: 2340 for the IgE assay. **A**) comparison of levels of CSP, MSP2, MSP3 and EXP1 antigens specific IgE in serum samples of comparator vaccine control or RTS,S vaccine from cohort 1. **B**) comparison of levels of CSP, MSP2, MSP3 and EXP1 antigens specific IgE in serum samples of comparator vaccine control or RTS,S vaccine from cohort 2. Blue represented “Protected/NE” and orange represented “Non-protected”. The results represent means ± standard errors (SE). Statistical significance was determined using Wilcoxon signed-rank test or Mann Whitney test; ns, not significant * *p < 0.05*, ** *p < 0.01* and **** *p* < *0.0001*. The horizontal lines indicate mean ± standard errors (SE).
