# Supplementary figures and images for: Blood Interferon Signatures Putatively Link Lack of Protection Conferred by the RTS,S Recombinant Malaria Vaccine to an Antigen-specific IgE Response
Source: F1000Res. 2017 Jul 18;4:919. Originally published 2015 Sep 29. [Version 2] doi: 10.12688/f1000research.7093.2 (PMC5580375; doi:10.12688/f1000research.7093.2)

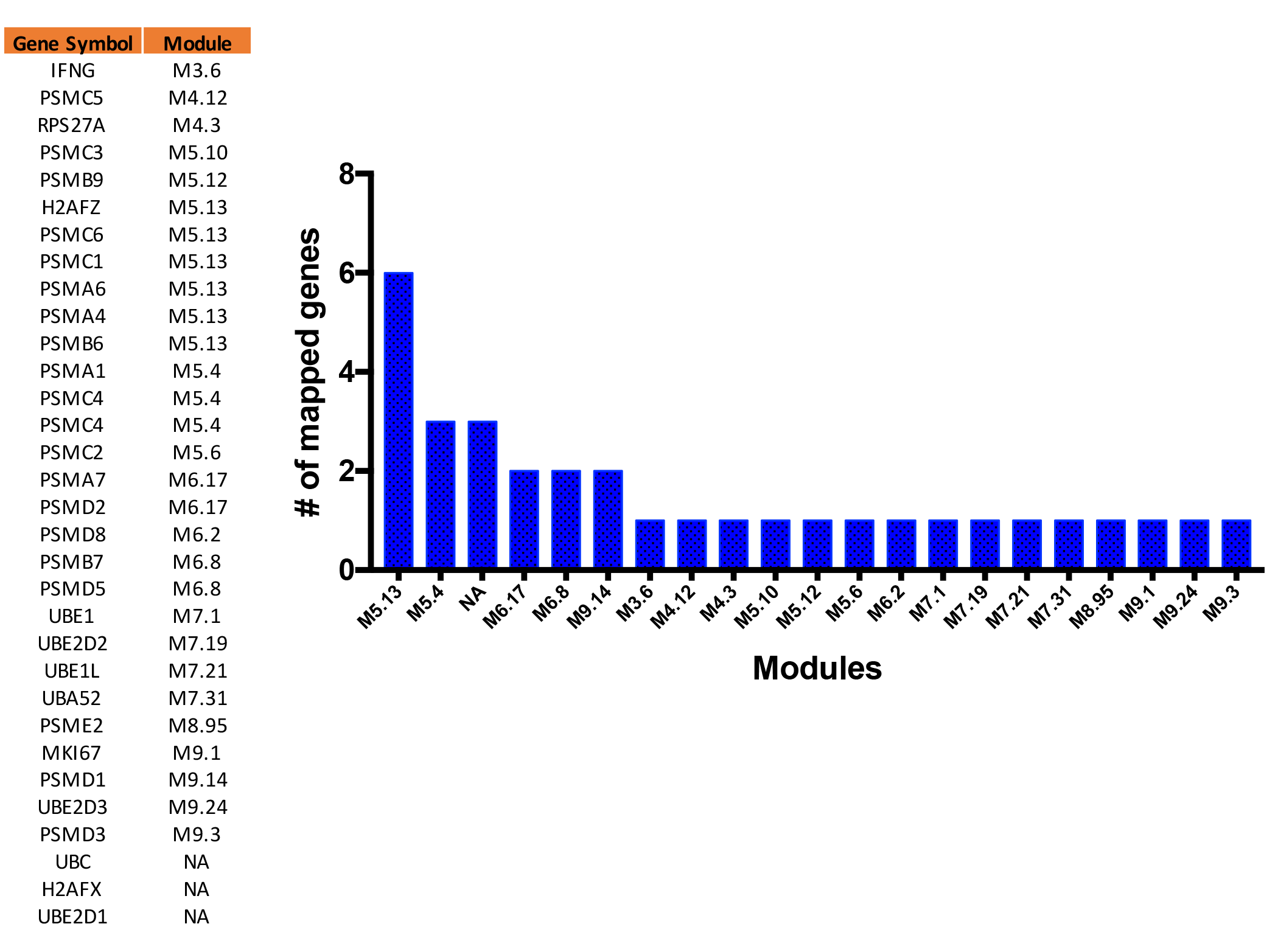

Supplement: Supplementary file 1 [file f1000research-4-13000-s0000.tgz › a33a4b33-f27e-4f45-8d13-7493ef75c2c3.tif]

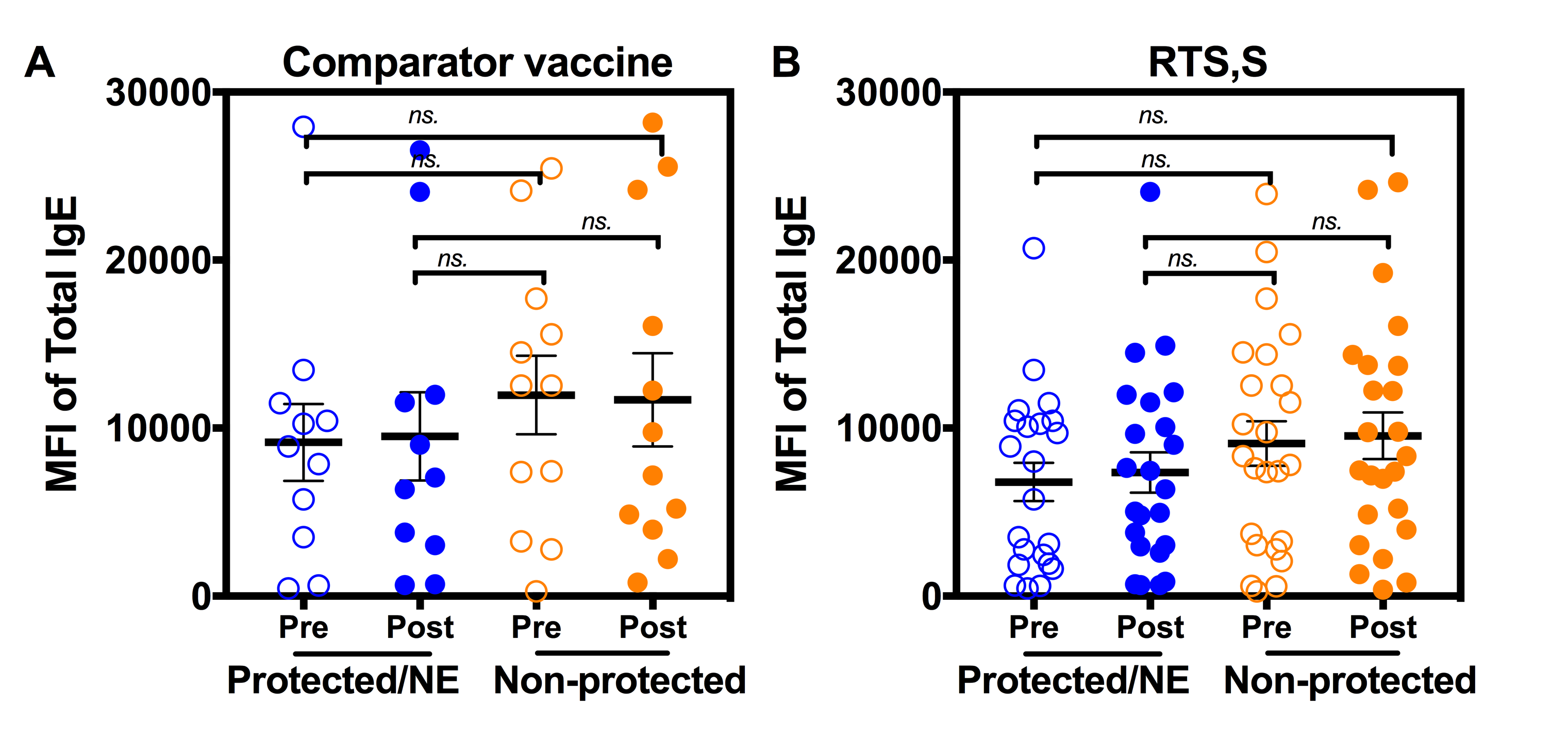

Supplement: Supplementary file 2 [file f1000research-4-13000-s0001.tgz › c5fb6baf-d251-4dea-a1d4-0d53ae579a05.tiff]

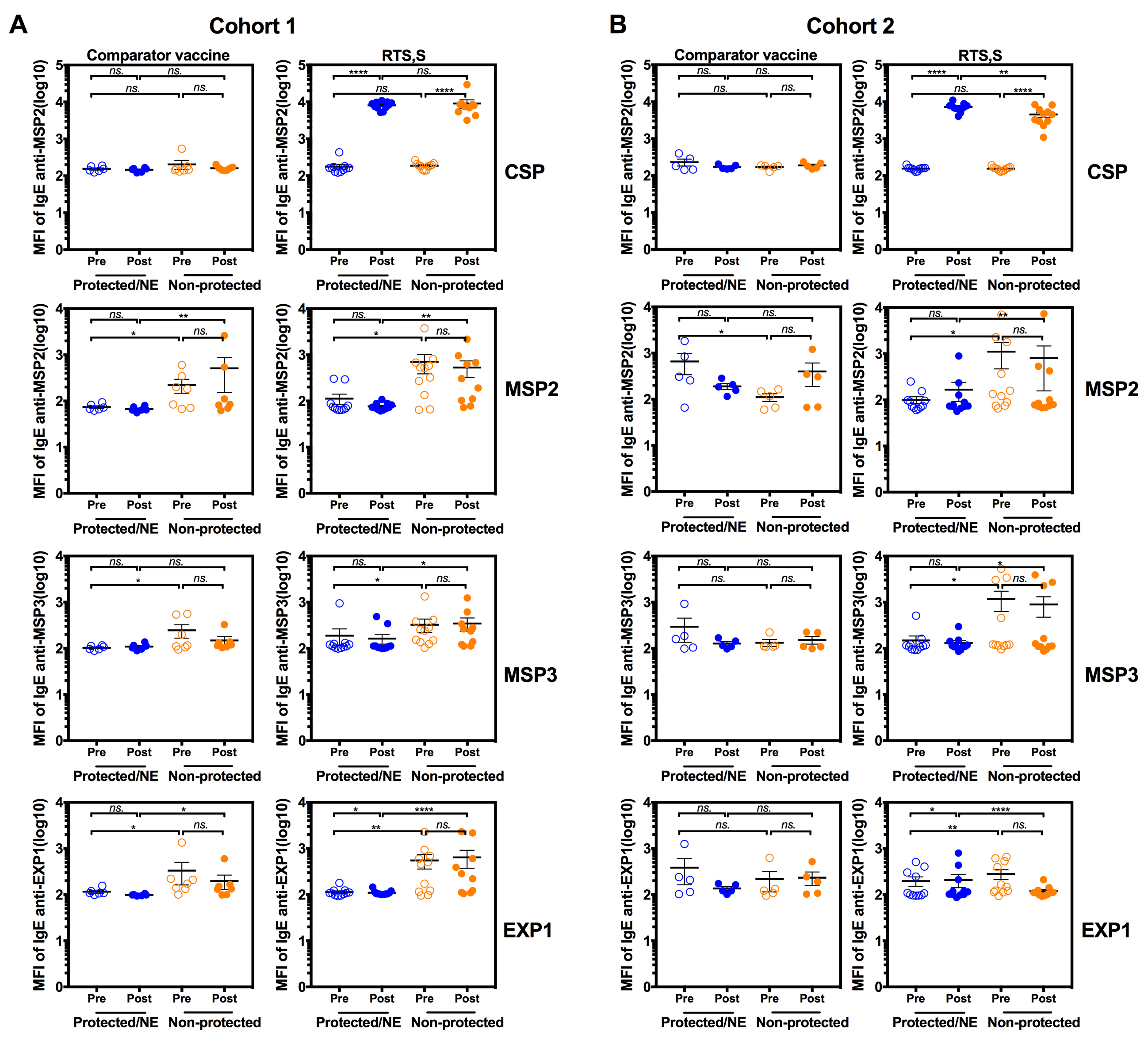

Supplement: Supplementary file 3 [file f1000research-4-13000-s0002.tgz › 6270a692-52ca-4ff4-8a74-0f6a6a474771.tiff]
