# Supplementary material for: Blood Interferon Signatures Putatively Link Lack of Protection Conferred by the RTS,S Recombinant Malaria Vaccine to an Antigen-specific IgE Response
Source: F1000Res. 2017 Jul 18;4:919. Originally published 2015 Sep 29. [Version 2] doi: 10.12688/f1000research.7093.2 (PMC5580375; doi:10.12688/f1000research.7093.2)
Supplement: Peer review history

# Peer review: Referee response for version 2

**DOI:** 10.5256/f1000research.13000.r24306

**Published:** 2017-08-29

**Authors:** Adrian J. F. Luty

**Affiliations:** 1 IRD MERIT UMR 216, French Research Institute for Development (IRD), Paris, France

I read with interest the newly revised version of the paper writen by Rinchai and colleagues. The revision is a clear improvement on the original submission. The authors have explored new avenues, generating new data that, in some cases, bolster their original conclusions concerning a potential immuno-modulatory role for vaccine-specific IgE responses. At the same time they acknowledge the fact that other, thus far unexplored, areas may reveal alternative explanations for the profiles they document, and that, perhaps most importantly, the results of immunisation in non-exposed North American adults - along with the associated immunological profiles - may prove to be a rather poor guide to the outcome of immunisation in sub-Saharan African babies.

I have read this submission. I believe that I have an appropriate level of expertise to confirm that it is of an acceptable scientific standard.

# Peer review: Referee response for version 1

**DOI:** 10.5256/f1000research.7637.r11338

**Published:** 2016-02-08

**Authors:** Adrian J. F. Luty

**Affiliations:** 1 IRD MERIT UMR 216, French Research Institute for Development (IRD), Paris, France

Over the last 3 decades or so the development and testing of candidate vaccines for malaria has seen numerous advances but an almost equal number of setbacks. RTS,S (aka Mosquirix) stands apart, for now, as the only such vaccine to have advanced as far as registration. Whilst that is clearly an achievement to be applauded, there nevertheless remain substantial gaps in our basic understanding of the immunological mechanisms involved in the protection conferred by the vaccine. The study reported here by Darawan Rinchai and colleagues represents an attempt to fill some of the gap in that knowledge.

The source data that Rinchai and colleagues were able to access for their analyses were derived from an original study authored by Kester and colleagues ^1^ that comprised a comparison of outcomes, including efficacy, of two formulations of RTS,S in different adjuvants administered to adult volunteers. Notably, that study documented higher titres of CSP-specific IgG as well as higher frequencies of 'activated' CSP-specific CD4 ^+^ T cells and IFN-γ-producing T cells in individuals who were protected from an experimental challenge infection with sporozoites compared to the study individuals who were not protected. The results of an adjunct to Kester and colleagues' study were published subsequently by Vahey and colleagues ^2^. That study documented gene expression analyses of peripheral blood mononuclear cells isolated from the volunteers at different time-points and identified - through the use of high-resolution geneset analysis - immunoproteasome processing of MHC peptides as predictively distinguishing protected from unprotected individuals.

Rinchai and colleagues' analytical approach employed a 'modular' method that they had previously developed 'in-house' and had used in other studies of vaccine-induced gene profiles. Here they used the same approach to assess gene expression profiles in the dataset generated by Vahey and colleagues. On that basis it would have been instructive for them to demonstrate the validity of their approach by assessing the immunoproteasome geneset identified by Vahey and colleagues in the context of its predictive capacity. Also, they need to justify the rationale for the module filtering procedure that discarded any displaying changes <15%. On what basis was that threshold chosen? In passing, for accuracy they also need to modify their repeated incorrect reference to 'blood' proteome to the correct 'peripheral blood mononuclear cell' proteome.

Rinchai and colleagues' principal finding concerning the association between upregulated expression of an 'interferon module' response and protection conferred by RTS,S appears to be consistent with the enhanced T cell responses associated with protection reported by Kester and colleagues in the same individuals. It would also be consistent with the known role for IFN-α in enhancement of naive B cell differentiaton ^3^. My main concern in the context of their discussion and conclusions relates to their speculative interpretation for the under-expression/down-regulation of the interferon module response they recorded both 3 & 14 days after the third RTS,S immunization in non-protected individuals. They contend that that particular profile is most likely to be linked to an aberrant IgE rather than IgG response to CSP. They conclude that this would result in IgE-CSP immune complex-mediated cross-linking of IgE receptors on myeloid cells that would in turn mediate the type of down-regulation of pDC activity they observed in the non-protected individuals. Whilst this may indeed be a plausible explanation for their observations, it is far from being the only one, and the authors need to recognize this. Firstly, it is of possibly central relevance here that RTS,S is a hybrid molecule comprising the CSP sequence conjugated to a 226 amino acid stretch of hepatitis B surface antigen (HBsAg). The same portion of HBsAg is co-expressed in unconjugated form along with the conjugate such that the purified vaccine spontaneously forms virus-like particles. Importantly, HBsAg has been shown to inhibit IFN-α production by pDC ^4^, implying that the 'non-malaria' component of RTS,S could thus potentially be exerting some influence on the outcome of immunization in terma of the anti-malarial protection generated. Secondly, a role for IgG-CSP immune complex-mediated inhibition of IFN-α production could also be envisaged through their interaction with the inhibitory FcγRIIB receptor expressed on pDC ^5^. Thus, I feel, the authors need to modify their article to incorporate discussion of these alternative, but not necessarily mutually exclusive explanations for their findings. In the context of their preferred conclusion, I wonder if might it not also be a possibility that they re-interrogate their dataset to try to identify possible changes in relevant 'modules' related to IgE/FCERI expression levels?

Finally, I also suggest strongly that they remove references to and discussion of publications that have proposed a role for IgE in malarial morbidity. Disease manifestations due to infection with *Plasmodium falciparum*  result from pathophysiological events associated with the asexual blood stage multiplication phase, reference to which in the context of putative antibody responses to a component of a vaccine that targets the pre-erythrocytic sporozoite stage are entirely erroneous in my view.

In conclusion I would say that Rinchai and colleagues' findings certainly provide some pointers to elucidating the immunological mechanisms governing the capacity of RTS,S to induce protective responses in some individuals but not others. I would nevertheless add the rider that the vaccine's capacity for induction of immunological responses in malaria non-exposed adults in North America may be quite far removed from its capacity to induce responses in sub-Saharan African newborns or infants, many of whom could have had some exposure to the pathogen or its products already either *in utero *or in early life. Such exposures could plausibly modulate their capacity to respond to vaccines in general and to a malaria vaccine in particular.

I have read this submission. I believe that I have an appropriate level of expertise to confirm that it is of an acceptable scientific standard, however I have significant reservations, as outlined above.

## References

[1] KesterKECummingsJFOfori-AnyinamOOckenhouseCFKrzychUMorisPSchwenkRNielsenRADebebeZPinelisEJuompanLWilliamsJDowlerMStewartVAWirtzRADuboisMCLievensMCohenJBallouWRHeppnerDG: Randomized, double-blind, phase 2a trial of falciparum malaria vaccines RTS,S/AS01B and RTS,S/AS02A in malaria-naive adults: safety, efficacy, and immunologic associates of protection. *J Infect Dis*.2009;200(3) : 10.1086/600120 337-46 10.1086/600120 19569965

[2] VaheyMTWangZKesterKECummingsJHeppnerDGNauMEOfori-AnyinamOCohenJCocheTBallouWROckenhouseCF: Expression of genes associated with immunoproteasome processing of major histocompatibility complex peptides is indicative of protection with adjuvanted RTS,S malaria vaccine. *J Infect Dis*.2010;201(4) : 10.1086/650310 580-9 10.1086/650310 20078211

[3] GujerCSandgrenKJDouagiIAdamsWCSundlingCSmed-SörensenASederRAKarlsson HedestamGBLoréK: IFN-α produced by human plasmacytoid dendritic cells enhances T cell-dependent naïve B cell differentiation. *J Leukoc Biol*.2011;89(6) : 10.1189/jlb.0810460 811-21 10.1189/jlb.0810460 21233412PMC3100762

[4] ShiBRenGHuYWangSZhangZYuanZ: HBsAg inhibits IFN-α production in plasmacytoid dendritic cells through TNF-α and IL-10 induction in monocytes. *PLoS One*.2012;7(9) : 10.1371/journal.pone.0044900 e44900 10.1371/journal.pone.0044900 23024774PMC3443230

[5] FloresMChewCTyanKHuangWQSalemAClynesR: FcγRIIB prevents inflammatory type I IFN production from plasmacytoid dendritic cells during a viral memory response. *J Immunol*.2015;194(9) : 10.4049/jimmunol.1401296 4240-50 10.4049/jimmunol.1401296 25821224PMC4820833

# Response

**Published:** 2017-06-22

**Authors:** Damien Chaussabel

**Affiliations:** Sidra Medical and Research Center, Qatar

*We would like to thank the reviewers for their time and constructive feedback. In this revised version, we have added a new result section and figure showing FCER1 transcript profiles in vaccinated individuals, as well as newly generated IgE profiling data measured in plasma samples of children from malaria endemic areas who received the RTS,S vaccine or a comparator vaccine. We have included below our response to the points that were raised in the review.*

**Approved with Reservations**

**...**

Rinchai and colleagues' analytical approach employed a 'modular' method that they had previously developed 'in-house' and had used in other studies of vaccine-induced gene profiles. Here they used the same approach to assess gene expression profiles in the dataset generated by Vahey and colleagues. On that basis it would have been instructive for them to demonstrate the validity of their approach by assessing the immunoproteasome geneset identified by Vahey and colleagues in the context of its predictive capacity.

***Authors:*** *Indeed, this is an important point. We assessed the overlap of the proteasome signature reported by Vahey et al. with our modular genesets. Six genes overlapped with module M5.13 and three with module M5.4 (Supplementary Figure 1). However, neither M5.13 nor M5.4 were identified as being associated with protection, or lack of protection following RTS,S vaccination.*

*We added the paragraph below in the result section “ **Decreased interferon module response at days 3 and 14 correlate with lack of protection” **and show results in supplementary figure 1*

*“* *Notably, we found that the genes constitutive of M1.2, M3.4 and M5.12 do not overlap with the day 14 immunoproteasome signature (32 genes) described by Vahey et al (Supplementary figure 1). All but three of the 32 genes mapped to the modules constituting our repertoire. Six genes mapped to module M5.13 and three to module M5.4, the remaining 23 genes mapped to 18 other modules. However, none of the genes mapped to either of the three interferon modules identified as associated with protection by RTS,S vaccine at Day 1, 3 or 14 after the third vaccination. Notably, IFNG, which was part of the 32 gene signature is among the genes constituting M3.6, which is associated with NK cells/Cytotoxic activity.”*

Also, they need to justify the rationale for the module filtering procedure that discarded any displaying changes <15%. On what basis was that threshold chosen?

***Authors:*** *We have added the sentence below in the manuscript to clarify this point;*

***“*** *T* *his arbitrary cutoff is set at three times the false discovery rate used for multiple testing correction (5%), which allows effective filtering of false positive results.”*

In passing, for accuracy they also need to modify their repeated incorrect reference to 'blood' proteome to the correct 'peripheral blood mononuclear cell' proteome.

***Authors:*** *The manuscript was edited accordingly and reference now being made to “Peripheral blood mononuclear cell transcriptome….” Throughout.*

Rinchai and colleagues' principal finding concerning the association between upregulated expression of an 'interferon module' response and protection conferred by RTS,S appears to be consistent with the enhanced T cell responses associated with protection reported by Kester and colleagues in the same individuals. It would also be consistent with the known role for IFN-α in enhancement of naive B cell differentiaton ^3^. My main concern in the context of their discussion and conclusions relates to their speculative interpretation for the under-expression/down-regulation of the interferon module response they recorded both 3 & 14 days after the third RTS,S immunization in non-protected individuals. They contend that that particular profile is most likely to be linked to an aberrant IgE rather than IgG response to CSP. They conclude that this would result in IgE-CSP immune complex-mediated cross-linking of IgE receptors on myeloid cells that would in turn mediate the type of down-regulation of pDC activity they observed in the non-protected individuals. Whilst this may indeed be a plausible explanation for their observations, it is far from being the only one, and the authors need to recognize this. Firstly, it is of possibly central relevance here that RTS,S is a hybrid molecule comprising the CSP sequence conjugated to a 226 amino acid stretch of hepatitis B surface antigen (HBsAg). The same portion of HBsAg is co-expressed in unconjugated form along with the conjugate such that the purified vaccine spontaneously forms virus-like particles. Importantly, *HBsAg has been shown to inhibit IFN-α production by pDC*^4^, implying that the 'non-malaria' component of RTS,S could thus potentially be exerting some influence on the outcome of immunization in terma of the anti-malarial protection generated. Secondly, a role for IgG-CSP immune complex-mediated inhibition of IFN-α production could also be envisaged through their interaction with the inhibitory FcγRIIB receptor expressed on pDC ^5^. Thus, I feel, the authors need to modify their article to incorporate discussion of these alternative, but not necessarily mutually exclusive explanations for their findings. In the context of their preferred conclusion, I wonder if might it not also be a possibility that they re-interrogate their dataset to try to identify possible changes in relevant 'modules' related to IgE/FCERI expression levels?

***Authors:*** *These comments have contributed significantly to the discussion and interpretation of the IgE profiling data presented in this revised manuscript:*

*With regards to the first point made in the comment above:*

*“* *An alternative explanation is advanced by Dr Luty in his comments, who mentions the potential relevance of the use of a 226 amino acid stretch of HBsAg as a conjugate for the CSP antigen in the RTS,S vaccine. Indeed this molecule has also been shown to inhibit Toll-like Receptor (TLR)9-mediated IFN-α production by pDC [Shi B., et al., PLoS One. 2012], and “could thus potentially be exerting some influence on the outcome of immunization in term of the anti-malarial protection generated” [Luty AJF., Referee report., F1000Research 2015, 4:919]. This is indeed another possibility, which warrants testing of the inhibitory capacity of the protein fragment used in RTS,S vaccine formulation. Furthermore the cell populations and signaling pathways involved in elicitation and modulation of interferon responses by RTS,S vaccine would also need to be investigated. It was found for instance that, rather unexpectedly, the induction of interferon response by the trivalent influenza vaccine is mediated by immune-complexes rather than TLR engagement [O'Gorman WE., et al., Vaccine. 2014].* *”*

*With regards to the second point made in the comment above:*

*“At Dr Luty’s suggestion we also examined levels of abundance of FCER1 subunits transcripts in the RTS,S vaccine dataset used in our analysis ( **Figure 4**). Consistently with what is observed during acute malaria infection (GSE34404) [* *22949651* *],* *FCER1A and FCER1G levels were respectively increased and decreased one day post-RTS,S vaccination. However, notably, FCER1G levels remained significantly elevated at day 3 post-vaccination in non-protected individuals, while they decreased to baseline levels in protected individuals ( **Figure 4D**). Abundance of FCER1G transcript was also significantly elevated in a third dataset where changes were measured in patients during episodes of asthma exacerbation (GSE24745) [22316092]. Moreover, we also checked expression level of FCGR2A and FCGR2B in the same dataset and found that abundance of FCGR2A and FCGR2B were not different in subjects who were protected and in those who were not protected.”*

Finally, I also suggest strongly that they remove references to and discussion of publications that have proposed a role for IgE in malarial morbidity. Disease manifestations due to infection with *Plasmodium falciparum* result from pathophysiological events associated with the asexual blood stage multiplication phase, reference to which in the context of putative antibody responses to a component of a vaccine that targets the pre-erythrocytic sporozoite stage are entirely erroneous in my view.

***Authors:*** *The IgE profiling results that have been newly generated illustrate the point made by Dr Luty. It shows a weak association with elevated baseline levels of CSP-specific IgEs and subsequent malaria morbidity, but a much stronger association with MSP-specific IgEs. These findings make the point raised and citations in question all the more relevant. The discussion points have been modified accordingly:*

*“Further evidence pointing to the influence of IgE responses comes from the literature and from results of our preliminary investigation reported below showing a dependence on both stages and antigen specificity of IgE on outcomes of malaria infection in children from endemic areas. Perlmann et al. identified IgE as a pathogenic factor in malaria, with immune complexes contributing to excess TNF induction in PBMC in vitro ^26^. Mice deficient for the high affinity IgE receptor have shown increased resistance to blood-stage of parasite infection, specifically implicating FCER1 expressing neutrophils as pathogenic mediators ^27^. A more recent study has established a link between asthma and atopic dermatitis and delayed development of clinical immunity to P. falciparum ^28^. Notably, in addition to shifting cytokine balance by promoting IL-10 and TNF production, engagement of high affinity IgE receptors has been reported to critically impair phagocytic function of monocytes, a mechanism that is essential for the control of malaria infection ^29^. The antigen specificity of IgE responses that have been associated with enhanced malaria morbidity remains to be determined. It is of relevance to RTS,S vaccination, at least in naïve subjects, since the vaccine targets the liver stage rather than the blood stage of the parasite.”*

# Peer review: Referee response for version 1

**DOI:** 10.5256/f1000research.7637.r11692

**Published:** 2015-12-21

**Authors:** Geneviève Milon

**Affiliations:** 1 Immunophysiology and Intracellular Parasitism Unit, Institut Pasteur, Paris, France

It is a pleasure to provide my comments and highlight some issues in your well-conceived and well-conducted re-analysis of dataset generated and assembled by Vahey *et al*; this  latter study was conducted to investigate changes in transcript abundance in Peripheral Blood Mononuclear Cells/PBMCs prepared from blood sampled from human volunteers who were recipients of  three doses of the recombinant  RTS,S vaccine containing a protein named Circum Sporozoite Protein/  CSP. Of note the CSP is displayed at the surface of by the human- invasive 3D7 *Plasmodium falciparum *developmental stage population named the invasive sporozoite population.

  

The invasive properties of sporozoite population

- reflect maturation within the salivary glands of the blood-feeding *Anopheles stephensi*/ *A.s*
- and is transmitted when these blood-feeding anopheline mosquitos are
  1. either probing the skin.
  2. or sampling their blood meals,
- whether it is one or the other process it is coupled to  delivery  of   both saliva *A. stephensi* **saliva **molecules and sporozoites.

Your re-analysis did allow generating interesting data sets

- with the identification of an interferon transcriptional signature at day 1 post-vaccination, correlating with protection,
- as well as the identification of  distinct interferon transcriptional signatures at days 3 and 14 post-vaccination correlating, at these  time points,  with  reduced or lack of protection of study subjects from subsequent challenges with sporozoites delivered by insectarium reared-blood-feeding *Anopheles *hosting mature sporozoites in their salivary glands

When concluding your re-analysis and the putative model accounting for the contrasted interferon signatures monitored at day 1 and days 3 and 14 post RTS,S vaccination, please could you also consider another biologically sound variable , namely the  potentially contrasted ratio profile of the *Anopheles* saliva molecules-binding IgE/IgG: indeed you are aware that infants and adults who share durably the habitats of blood feeding *Anopheles* spp are more frequently exposed to *Anopheles* females that do not act as *Plasmodium* sporozoite vectors but that deliver saliva in the dermis a site where many saliva derived agonists are rapidly sensed by immune sensors the T cell receptors and the membrane Ig receptors included; such features are reflected by the ability to detect saliva –reactive T cells and saliva-reactive Ig molecules in the blood at very early times points post either skin probing or blood sampling by blood feeding insects.

Please note that blood- feeding *Anopheles *does not transmit malaria – the latter term is expected to depict only the symptoms that occur over the asexual *P. falciparum* intra- erythrocytic developmental program : the blood feeding Anopheles delivers saliva while transmitting  sporozoites that will further reach the hepatocytes where proceeds  the generation of *P. falciparum*  merozoites that are invasive for erythrocytes .

Please could you review the Fig 3 legend:

**" *Figure 3. Proposed immunological mechanism determining protection – or lack thereof – in adult human individuals to whom was inoculated the RTS,S vaccine.***

*Our model infers that the interferon signatures observed on days 1, 3 and 14 post-vaccination correlating with outcome of the processes that deploy post the co-delivery of Anopheles saliva and P. falciparum sporozoites, are the result of engagement of Fc receptors by immune complexes.*

*According to our model no interferon signatures should be observed following administration of the first vaccine dose of the CSP- containing RTS,S vaccine, in absence of pre-existing immune effectors/regulators reactive to P. falciparum Circum Sporozoite Protein/CSP.*

*The injection of the first two doses of vaccines should elicit a humoral response, which in non-protected individuals is dominated by CSP- binding IgE rather than IgG. CSP- Ig- immune complexes should form when the third vaccine dose is administered.*

*The transient interferon response elicited in individuals who develop a protective response and that we observed at Day 1 could be mediated ****by engagement of the FcγR by IgG-CSP immune complexes as has been described earlier in the context of influenza vaccination) ^22^.*

*Our model predicts that IgE-CSP complexes form in non-protected individuals and cross-link the high affinity IgE receptor at the surface of cells of  the myeloid lineage. FCER1 engagement would in turn mediate reduction in levels of IFN-inducible (IFI) transcripts that is observed on days 3 and 14.*

*This ****reduction of constitutive levels of IFN inducible transcripts in the non-protected group would be countered at least partially on Day 1 by residual IgG-IC response in subjects displaying mixed IgE/IgG humoral responses.*

*If this is indeed the case the levels of M1.2 reduction on Day 1 in individuals displaying CSP-binding IgE should be inversely correlated with levels of CSP-specific IgG."*

Many thanks for your attention

I have read this submission. I believe that I have an appropriate level of expertise to confirm that it is of an acceptable scientific standard.

# Response

**Published:** 2017-06-22

**Authors:** Damien Chaussabel

**Affiliations:** Sidra Medical and Research Center, Qatar

We thank you very much for your time and valuable comments.

 All your edits have been incorporated in the legend of Figure 3. 

 At the end of the conclusion we added the following sentence to reflect the suggestion that you have made, which is made all the more relevant by the data that we have generated and are now presenting in the second version of this manuscript.

*"Furthermore, as pointed out by Dr Milon in her comments, extending the investigation to include profiling of IgE which are specific for antigen present in the saliva of the vector is also warranted [REF]."*

*REF: Milon G. Referee Report For: Blood Interferon Signatures Putatively Link Lack of Protection Conferred by the RTS,S Recombinant Malaria Vaccine to an Antigen-specific IgE Response [version 1; referees: 1 approved, 1 approved with reservations]. F1000Research 2015, 4:919*
